# Neural Representation. A Survey-Based Analysis of the Notion

**DOI:** 10.3389/fpsyg.2017.01458

**Published:** 2017-08-29

**Authors:** Oscar Vilarroya

**Affiliations:** ^1^Departament de Psiquiatria i Medicina Legal, Universitat Autònoma de Barcelona Barcelona, Spain; ^2^Institut Hospital del Mar d’Investigacions Mèdiques (IMIM) Barcelona, Spain

**Keywords:** hippocampus, MVPA, mental representation, neural representation, place-cell research, representation

## Abstract

The word representation (as in “neural representation”), and many of its related terms, such as to represent, representational and the like, play a central explanatory role in neuroscience literature. For instance, in “place cell” literature, place cells are extensively associated with their role in “the representation of space.” In spite of its extended use, we still lack a clear, universal and widely accepted view on what it means for a nervous system *to represent* something, on *what* makes a neural activity a representation, and on *what* is re-presented. The lack of a theoretical foundation and definition of the notion has not hindered actual research. My aim here is to identify how active scientists use the notion of neural representation, and eventually to list a set of criteria, based on actual use, that can help in distinguishing between genuine or non-genuine neural-representation candidates. In order to attain this objective, I present first the results of a survey of authors within two domains, place-cell and multivariate pattern analysis (MVPA) research. Based on the authors’ replies, and on a review of neuroscientific research, I outline a set of common properties that an account of neural representation seems to require. I then apply these properties to assess the use of the notion in two domains of the survey, place-cell and MVPA studies. I conclude by exploring a shift in the notion of representation suggested by recent literature.

## Introduction

The word “representation” (as in “neural representation”), and many of its related terms, such as “to represent,” “representational” and the like, play a central explanatory role in neuroscience literature. For instance, in “place cell” literature, place cells are extensively associated with their role in “the representation of space.” Lately, the notion of representation has gained more visibility since the development of new methods that try to translate brain mapping markers (such as BOLD signals) into stimuli features, and vice versa. Indeed, the development of methods such as multivariate pattern analysis (MVPA), which attempts to identify the contents of brain activity, has put the notion of representation in the center of the debate.

All of these uses seem to be anchored upon a clear and accepted conception of “representation,” at least in the domain of neuroscience. However, as has already been stated, “put briefly, though there is a vast quantity of on-going research dependent on representations, and though there is a vast quantity of ongoing research *on* representation, no scientist knows how representations represent” (Dietrich, 2007, p. 1, italics in the original). Indeed, we still lack clear, universal and widely accepted views on what it means for a nervous system *to represent* something, on *what* makes a neural activity a representation, and on *what* is re-presented.

A bibliographic search for the term “neural representation” yields a wealth of papers using the term but very few addressing the notion itself or examining its properties, implications, or theoretical models. The few of them that do meet such criteria are not recent (DeCharms and Zador, 2000; Markman and Dietrich, 2000a,b; Edelman, 2003). It is true that if we look for a related term, such as “neural code,” the literature indeed provides theoretical treatments of the notion in abundance. However, the notion of neural code concerns only a part of the notion of neural representation: the way a neural representation holds information. More importantly, it also assumes that neural representations are based on handling information in a codified manner. There are, however, other views of neural representation that do not require a neural code, such as, for example, dynamic systems approaches (Eggermont, 1998). Hence, focusing on the neural code will beg the question that I intend to address here: that is, examining the notion of neural representation itself.

The lack of a theoretical foundation and definition of the notion does not seem to have any impact on actual research, after all. As with many other terms, such as circuit, area, process, system, or network, we need not define it every time we use it nor give a specific reference for the term to be able to use it properly. In fact, there seems to be a conventional wisdom about the notion (as with all other basic widely used and not-defined terms) that has allowed authors to use the term without worrying much about details.

In this sense, whether or not it is useful to analyze the notion of “neural representation” is a legitimate question, as researchers use the term without having a working model of what it corresponds to and yet still produce relevant scientific work. However, in my opinion, the project is both legitimate and useful. The notion of “neural representation” is central to neuroscience, because, in the majority of studies, it corresponds to “what is to be explained,” such as, characterizing what a spike train corresponds to, and or “how we explain it,” by using representations to explain, for instance, how animals navigate efficiently. In short, the notion of neural representation plays a central role in explanations of the neural mechanisms underlying brain function. This means, among other things, that assuming a particular notion of representation constrains the questions one asks experimentally, the design of the experiments themselves and the interpretation of the results. If scientists can do without a clear idea about the notion, it is not because it is absent from their work, but because they implicitly assume a particular model of the notion. Hence, in my opinion, exploring such implicit models of the notion, and what they imply, is necessary and timely. To consider that the brain can code for objective features of the environment such as an Euclidean coordinate map of geographical space, or to consider that the brain cannot code for objective features of the environment point to quite divergent models of brain function. The experimental questions, designs and interpretations of assuming one or the other approach develop completely different frameworks. Therefore, in my opinion, it is critical to understand the model of “neural representation” that neuroscientists assume, and to characterize what the model implies empirically.

Such is my aim here: to carry out a model-dependent analysis of “neural representation,” that is, an analysis whereby the experimental findings in neural-representation studies are supposed to fit the theoretical model derived from the authors’ replies, and the literature on the topic. Specifically, I first show the results of a survey of authors of empirical studies in two domains—place-cell and MVPA research. This allows me to identify what such authors have in mind when they use the notion. Based on their replies and on a review of their research, I outline a set of common properties that an account of neural representation seems to require. I then apply these properties on the two domains of the survey: place-cell and MVPA studies. I conclude with a speculative note based on recent theoretical approaches.

## Survey

I carried out an online survey of authors of studies in two domains: place-cell research and MVPA research. These two domains examine neural activity at very different levels of analysis, using very different methodologies and techniques, but sharing the notion of “neural representation” as a central element of their research. On one hand, under the label of “place-cell research,” I classify studies that focus on the firing characteristics of hippocampal neurons in awake and free-moving animals. These sorts of studies has provided data associating environmental stimuli and behavior with neuronal activity. The electrophysiological data capture a number of different dynamics of activity and coherence among groups of cells, including “place cells,” which have a preferential spatial location and “grid cells,” which produce a preferential periodic array of locations’ activity, as well as other neurons associated with spatial activity, such as “head direction” or “border” neurons. On the other hand, under the label “MVPA research,” I include studies that use multivariate statistical tools, known as classifiers, to characterize the encoding and decoding of environmental features in BOLD-based activation patterns. Both field studies share the aim of understanding how a brain makes sense of the world based on a comprehensive model that encompasses cellular, neural systems and behavioral data using different methodologies and units of analysis.

### Methods

#### Selection of Authors

I identified candidate studies by searching for peer-reviewed publications published between 2010 and 2015 in Scopus. For MVPA research, I used the following search string:

PUBYEAR > 2009 AND PUBYEAR < 2016 AND TITLE-ABS-KEY (“fMRI”) AND TITLE-ABS-KEY (“represent^∗^”) AND (TITLE-ABS-KEY (“MVPA”) OR TITLE-ABS-KEY (“multivoxel”) OR TITLE-ABS-KEY (“multi-voxel”))

For place-cell research, I used the string:

PUBYEAR > 2009 AND PUBYEAR < 2016 AND TITLE-ABS-KEY (“place cells”) AND TITLE-ABS-KEY (“represent^∗^”)

The searches produced 231 papers for MVPA research and 250 for place-cell research. I then scanned each paper for the stem “represent^∗^,” and selected only those studies that used the notion “representation” in the general intended meaning of “standing for” some feature or stimulus in the world, in contrast to when it was used in the sense of “portraying,” such as, “Figure 1 represents the position of the cell...”.

From the selected set of publications, I identified the corresponding authors and removed authorship duplication, resulting in 266 papers in total, corresponding to 132 MVPA authors and 134 place-cell authors.

#### Questions

The online survey consisted of an email directed to the corresponding author with the subject “Research Query” and the following text:

∗ What is your intuitive definition of representation? (I am especially interested in your intuitive approach, because I believe it might reveal a richer picture of the notion than the one offered by academic definitions)
∗ In your opinion, can representation be measured?
∗ If so, what would be the measure of representation (mutual information, reliable correlations…)?
∗ What unit of analysis should be used (cell, local population of cells, networks…)?

The answers of all the authors will be treated anonymously. At most, if the survey is eventually published, I might include, for illustrative purposes, some literal definitions, without indicating its author.

I introduced the preference of intuitive definitions, because the survey was intended to reveal the actual uses of the notion and to explicitly avoid the temptation of relying on dictionary definitions. Additionally, I decided to include some examples, such as “mutual information” or “local population of cells,” because in a pilot survey that I had carried out among colleagues of mine who did not participate in the survey, I found that readers had some doubts about what I was referring to as “measures of representation” or “levels of analysis.”

The emails were sent from March 28, 2016, until April 8, 2016. There were 102 responses (38.4%) that included replies to the questions and 14 refusals (5.2%), mainly attributed to a lack of time. There were 150 (56.3%) emails that went unanswered.

This study was carried out in accordance with the recommendations of the Comissió d’Ètica en l’Experimentació Animal i Humana (CEEAH) at the Universitat Autònoma de Barcelona. Consent was obtained by email from the survey participants.

## Minimal Definition

First, the authors’ replies reflected a general concurrence that a general theory of neural representation was lacking. As one author put it bluntly: “We don’t know how the brain represents.” However, there are some basic aspects of the notion about which there seemed to be some sort of agreement.

Regarding the question of definition, there likely were as many definitions as there were authors’ replies; however, all but one of the authors endorsed the idea that neural representation demands two components: a neural component and an environmental component. The neural component is usually referred as a “pattern of neural activity” or simply “neural activity,” as well as related terms such as “neural substrate,” “neural response,” or “spike activity.” Only fifteen authors did not use a neural term to refer to the internal component. I will return to this point below, upon describing the different conditions for neural representation. For the environmental component, there was a wider variety of terms, among which we could determine three main semantic domains. First, there were those authors who used expressions such as “external world,” “outside world,” and “the environment;” second, there were those authors who used terms such as “stimulus,” “stimulus features” or “stimulus properties;” and, finally, there were those authors who used terms such as “behavior,” “state of the animal” or “dimension of experience,” although in this case, the qualification of “environmental component” is less clear, since it could include internal elements such as, “hunger” or “pain,” for example.

Regarding the type of relationship that exists between neural activity and the environmental component, the authors seemed to recognize that “representing” involves a special relationship between both components by which the neural component *stands for* the environmental one. The exact wordings of the replies could be further classified into four different, but probably not incompatible, approaches. First, there are authors who used verbs such as “point” or “designate.” These authors seemed to embrace a neutral position in which the representational relation would only be defined by the connection between the neural component and the representee, leaving open the specifics of the relation. Second, there were the authors who defended the idea that to represent is for a neural activity to carry information about the environmental feature that the neural activity is supposed to represent. This description corresponds to the model suggested in the few extant theoretical models of neural representation: “a representation can be understood as a signal that is used in a transformation of information” (DeCharms and Zador, 2000). Note, however, that we have two very different types of information that are used interchangeably in neuroscience. On one hand, we have Shannon information, which is a measure of the efficiency of the communication channel. The brain is sometimes treated as a communication channel, and information is used as measure of the statistical dependency between a signal—say, a specific firing rate—and certain stimuli properties. This is the usual use of the notion in place-cell research. On the other hand, information can be used in the sense of “semantic information”—that is, as the content of what a neural representation is supposed to be causally, or at least reliably, correlated. In contrast to Shannon information, semantic information is not the measure of the statistical relationship between the thing and the neural representation; rather, it is the thing (e.g., a rabbit or a banana) that the representation is about. This is the usual use in MVPA research. Both types of information are not incompatible, but they correspond to different measures; therefore, they should not be used without specifying what sort of information is assumed. Neither the replies of the authors nor extant hypotheses about neural representation clarify which type of information should be the one to take into consideration. We will have to leave the issue unresolved for the moment.

Third, there were those authors who used verbs such as “encode” or *“*code.” In doing so, they seemed to characterize the representational relation as one involving some formal correspondence between the constituents of the neural pattern and the environmental feature. The neural codes that have been proposed to date have been based on the frequency and timing dimensions of a spike train. However, despite the large number of studies and models that have studied the neural code, we still do not know what code the brain uses and whether it is at the basis of neural representations.

Finally, we have those authors who used verbs such as “map,” “model,” “mirror,” “picture,” or “copy.” These authors seem to characterize the representational relation as one based on a rationale of isomorphism between neural representations and their representees. In other words, representations would be representations because they *resemble* their representee. Isomorphism is a loose notion that suggests a structure-preserving relation between the representation and the representee, such as the relation by which a map resembles a territory because the map holds a spatial resemblance with the territory. Among others, the possible topographical representations of space in the hippocampus, the results of mental rotation experiments and MVPA mind-reading studies based on similarity measures have all suggested that neural representations might implement certain structure-preserving relations with their respective representees. The problem with isomorphism, however, is that it can be arbitrarily applied in so many ways that it can ultimately undermine its explanatory role. The fact is that we can potentially establish arbitrary mappings between any two patterns if we are patient enough when looking for a suitable isomorphism (Putnam, 1988). Therefore, even if the authors using this set of terms can be grouped together, one cannot be sure whether the sort of isomorphism they have in mind is equivalent.

The four semantic domains that I have proposed to distinguish among the authors’ replies have to be taken with a pinch of salt. It would indeed be misleading to take the use of the terms “encode,” “carry information,” “map,” as absolute markers of a particular theory. Moreover, as has already been noted, there are still no reference theories against which to contrast the use of these terms; hence, we cannot be sure whether the authors were truly implying what I have suggested in my summaries. More importantly, the four different approaches are not, in principle, incompatible with each other; consequently, they could eventually be subsumed into a common model. Moreover, I believe we can provide a minimal definition that could be supported by the majority of the authors, namely:

A neural representation is a pattern of neural activity that stands for some environmental feature in the internal workings of the brain.

Even if this definition reliably characterizes a minimal shared conception of neural representation among active researchers, it still holds true that authors use the notion regardless of not having any theory of neural representation available. However, as written above, this has not been a hindrance. In fact, neuroscientists overcome the lack of a theoretical framework by a number of implicit or explicit assumptions about how to characterize instances of neural representations. This has allowed neuroscientists to yield a large and homogeneous corpus of empirical studies, regardless of the domain of study.

Is it possible to extract these assumptions from the survey and from their actual research? I think that the answer is in the affirmative, and in what follows, I will present the set of properties that can be seen to configure common criteria for building empirical accounts of neural representation. For reasons of clarity, I will address these properties within the three main components of any account of neural representation: (a) the pattern of neural activity; (b) the environmental representee; and (c) the association between the two. I will address each one in turn, and I will then use them to assess the two domains of focus in this paper: MVPA and place-cell research.

## Criteria for Neural Activity

As I indicated above, the most common idea among authors was that neural representation corresponds to some pattern of neural activity. Nevertheless, the authors seemed very flexible and pragmatic in accepting what type of neural activity might underlie a certain neural representation. For the unit of analysis, only one author considered that the notion of representation belongs exclusively to the conscious level, whereas the rest adopted a pragmatic and comprehensive approach addressing how the attribution of representational properties is a contextual criteria that depends on the feature or stimulus to be represented: different features are represented in different levels of analysis, from cells to networks. Among the different replies, such expressions as the following were common: “any level of analysis can work (cells, networks, behavior),” “all analysis levels of the organization of the nervous system can be considered pertinent,” “the unit of analysis is not fixed, functional roles can be attributed at any descriptive level,” “representations can occur in single cells, small networks, and across the entire brain,” and “the unit of analysis can again be anything, as long as is clear that any claims are also made on the level of analysis.”

In contrast, with the flexibility found relative to the type of neural activity considered to reflect neural representation, the authors and extant literature have been quite strict in the operational and formal features that any candidate should exhibit. I have summarized the features that a pattern of neural activity must exhibit to be endorsed as a neural representation in the following three conditions:

(i) A pattern of neural activity must be **well-formed**.
(ii) A pattern of neural activity must be **self-contained**.
(iii) A pattern of neural activity must be **neurally sound**.

The first condition required of patterns of neural activity is for them to be “**well-formed.”** I have borrowed the notion of “well-formedness” from the domain of linguistics. In linguistics, a well-formed sentence is one that is judged to be correct according to the set of rules and constraints of the grammar under scrutiny. In transferring the term to the domain of neural representation, the idea is that the representational competence should be based on a specific “grammar” that explains how constituent parts must be formed, as well as what rules and constraints should be followed to build each particular pattern of neural activity. More importantly, the grammar also explains how a particular pattern, based on the combination of the basic features of the neural activity, relates to specific features of a particular representee. For instance, in the case of place-cell research, the grammar of the representational roles is based on such features as timing (e.g., latency and duration) or spike rate. Note, however, that at present, the grammars suggested for place cells or MVPA patterns are merely hypotheses, because we still do not know the codes that are actually used by the brain. In any event, extant grammars are sufficient to systematically identify a specific pattern of neural activity as a possible neural representation and to distinguish it from other neural activities that are considered—for instance, noise.

Regarding the second condition, by “**self-containment**,” I mean the property of being able to fulfill the representational role regardless of whether the representee is present. The self-containment condition is a fundamental property for neural representation. Classically, it has been assumed in all representational models of cognition (Johnson-Laird, 1983; Pylyshyn, 1984; Pinker, 2004), in more modern approaches to neural representation (Wilson, 2002; Grush, 2004), and even in discussions of the representational capabilities of the hippocampus (Wamsley and Stickgold, 2010; Mayford and Reijmers, 2016). The fact is that without self-containment we cannot properly talk about representation. As one author phrased it, “We think of neural representations as a pattern of neural activity that allows a subject to re-experience the original stimulus even when it is not physically present,” whereas another reply maintained that “the same pattern of activity can then be used for planning or retrieval of memory about the previous experience.” Indeed, a basic role of a representation is precisely to substitute the representee in the internal workings of the brain and allow thinking, planning or imagining about the representee without the representee being present. The self-containment of neural representation requires that the pattern of neural activity be capable, unto itself, of substituting the content, causal roles and inferential processes of the representee in the internal workings of the brain. The assessment of self-containment is not straightforward, because we need an independent assessment that the neural representation works as a substitute of the environmental element, and this might be outside the scope of present neuroscientific methodology. However, it can be indirectly assessed by means of the very same methodology employed to identify neural activity. For example, one can assess such an assumption by activating the particular representation under scrutiny, without the representee being present, and prove that it contributes to inferential processes in the same way as would occur if the representee were present.

Finally, we have the **neurally sound** condition. By “neurally sound,” I understand here that any candidate for neural representation must be based on a sound account of how the measure employed (BOLD signal, spike activity, etc.) is a correlate of the representational activity. This is an implicitly held assumption that can be derived from the authors’ replies or extant neuroscience. For example, as one author put it: “We obviously need first to understand how our variables correspond to the neural activity that underlies a particular representation.” More specifically, as another author maintained: “What we call neural representation based on our measurement techniques may have little or nothing to do with how relevant information is actually being processed in the brain.” For one thing, the development of new techniques and methods of revealing brain activity results in a number of measures that are correlated to neuronal activity. However, the connection of these correlations to the actual neural activity that underlies the suggested representation is in some cases still a matter of discussion. The problem is that without a sound account, research runs the risk of losing sight of what the variables really measure. Therefore, the need for a justifiable rationale of the neural basis of the approach is especially necessary.

## Criteria for Representees

By “representee,” authors tend to refer to some element of the environment of the individual. Common to what all authors included in such a category is some mention of stimuli, in general, or of particular features or properties of relevant stimuli. The fact is that there is no special choice in the category or scale for a representee; it can be an object, the slope of a line, a behavior, a place, etc. The sole inclusion criteria that authors seemed to support for representees are that the candidate must be relevant for the studied organism and that the organism must be in possession of mechanisms that can detect such a representee. Categories and scales notwithstanding, the authors’ replies and extant research have suggested that there are certain requirements that the representee has to satisfy to be accepted as a suitable candidate. I have summarized these requirements in the following properties:

(i) Representees must be **measurable** magnitudes.
(ii) Representees must be **self-subsistent**.
(iii) Representees must be **meaningful** for the organism in question.

Let us address first the **measurable** requirement, which states that the representee must be some type of physical magnitude present in the environment (DeCharms and Zador, 2000). Extant research and authors’ replies recognize that magnitudes can correspond to a wide variety of environmental phenomena at different categories and scales, including objects, behaviors, places or properties, with the only condition that they be quantifiable by measurement. This is particularly relevant because neuroscientists often focus their study on features that are not physically quantifiable magnitudes themselves but are supervenient on physical quantities. For instance, color is a feature of objects that is usually studied as the content of representations; however, what one perceives as color is not a quantifiable property in itself but the outcome of the processing of different physical properties of the object, as well as of other elements. Color perception depends on the source of light illuminating the object, the spectrally selective reflectance of that light by the object, the surface of the object, the illuminating variability in the scene, and certain contingencies in the neural activity that mediates visual experience. In this case, even if we will consider “color” as the feature of study, the physical magnitude that will have to be considered the representee is the set of physical magnitudes of objects that can be used to represent color perception. Once a first order of representees is established, even then it would only be possible to talk about the neural representation of “color” if the proposal were also to explain how first-order neural representations are processed to yield a neural activity that could correspond to the neural representation of color. Another example would be subjective or abstract variables, such as beauty. Likewise, with color, abstract or subjective variables would have to be transformed into objective measurable magnitudes to be characterized as a representee. Beauty, for example, could be transformed into a set of operational properties (e.g., symmetry) that could be quantified.

The **self-subsistence** condition for representees is the symmetric condition of the self-containment of neural representation. Indeed, if a representation is a relation between two components, these two components must be independent; otherwise we would not be able to establish a *relation*. Therefore, we must be able to detach and characterize the representee independently from the neural activity. As one author put it, “the thing represented must be a distinct element of the world.” This is essential for representations. Representations are conceived as the building blocks of knowledge because they provide an explanation of how we know a piece of the world—by *representing* it—but they also provide an explanation of how we do not know a piece of the world—by *not representing* it—as well as an explanation of how we wrongly believe to know a piece of the world—by *misrepresenting* it. Indeed, the nature of the representational function implies that the relation can *malfunction*: the organism can misrepresent something in the world as something else. Hence, if the representation is supposed to truthfully represent some component of the world, we, as independent observers, must be capable of saying whether the component of the world (a fruit, a form or a place) is there or not. We must be able to say, “there is a rabbit,” “there is no rabbit,” or “there is a fox, as a basis for asserting,” “the organism has correctly represented the rabbit as a rabbit” or “the organism has misrepresented the fox as a rabbit.” Therefore, the representee must be autonomously characterizable; otherwise, the representee cannot be represented at all.

Finally, we have the **meaningful** requirement. The issue of meaningfulness is widely accepted as critical and has been subjected to very extensive analyses. In general, by “meaningful,” authors seem to suggest that the representee has to have a certain biological value (adaptive, ecological, etc.) or individual sense for the organism. For instance, some replies concurred with the idea that representees can only correspond to “meaningful dimensions of experience (e.g., location),” while another maintained that “whether or not (…) a ‘representation’ [in the sense of representee] has any relevance to the operation of that brain requires further empirical enquiry [than simply attesting the association]. Some ‘representations’ will be merely epiphenomenal, indicating something to the experimenter that has no direct relationship to the operation of the brain.” Hence, the need for this condition comes from the fact that the environment offers an open set of ways of being characterized, and only a determinate sub-set of those are the ones that organisms use as sources of knowledge. These elements are therefore the ones that are supposed to be “represented.” The problem is that the meaningfulness of the environment for a particular organism is not a given. If the scientist selects some piece of the environment because she simply believes it is relevant, without independent evidence apart from an educated guess, she runs the risk of establishing a spurious correlation. As I indicated in the case of the neurally sound condition, the versatility of the new techniques and methods of correlation between brain activity and environmental magnitudes makes it relatively easy to find correlations for reasonable ways of segmenting the environment, regardless of its meaningfulness for the organism under study. For example, assume that a scientist believes that “things that weigh less than 125 g” is a possible representee. Moreover, assume that she finds a neural component that reliably and solely correlates with “things that weigh less than 125 g” and that such a neural component complies with the well-formed, neurally sound and self-containment requirements. Then, if we do not introduce the meaningful condition, she will be licensed to consider it to be a good candidate for representee. Of course, it could certainly be that “things that weigh less than 125 g” would be a meaningful representee, but the point here is that the choice of the representee must be based on as much independently based evidence as possible. The choice of a representee should take into account its biological value to the organism for that particular type of situation or function, the sensory and cognitive endowment of the particular organism, and the developmental stage of the organism, as well as other factors. In very simple organisms, meaningfulness can be associated with feeding, protective or reproductive elements. Obviously, as we get to more complex organisms, the notion of meaningfulness becomes more loose and, therefore, more difficult to assess. In general, though, we could say that the piece of the environment that has meaning must be inferred from the way the organism responds to such an element, that is, attesting that it has causal consequences in the behavior of the individual. In contrast, if a particular piece of the environment is singled out but has no impact on the behavior of the individual, then it would be advisable to avoid it.

## Criteria for the Representational Relation

Any account of neural representation is based on the *relation* between the patterns of neural activity and specific representees. It is the representational relationship that defines an account of representation. As we have seen, though, we still lack a general theory of neural representation and, logically, of the representational connection. Therefore, it would also seem difficult to identify how specific empirical accounts of representational associations should look. However, as we have seen, neuroscientists seem to overlook this difficulty by assuming that the representational relationship exists and that their task is to identify a possible relation between two components, regardless of the nature of the relation in question.

In general, the different representational approaches (MVPA and place cell research) use statistical measures to identify relations between neural activity and environmental magnitudes. Indeed, this type of statistical correlation is the basis for nearly all uses of representation in neuroscience. Furthermore, according to the survey responses, the authors were very flexible with regard to the specific statistical measures used. The survey and extant literature seem to show a pragmatic flexibility regarding the ways of identifying and characterizing the representational association. Generally, any measure that identifies a correlation between a particular representee and a particular pattern of neural activity is acceptable, including mutual information, similarity analysis and the like. Common among the replies were expressions such as the following: “Any measure that determines the degree of mutual information can be used” or “representation can be measured in many ways, including both linear correlation and information theory measures.”

The only condition that the survey and extant literature have seemed to ask that a representational correlation show is for it to be **reliable**. I understand this term “reliable” to refer to the methodological condition of showing that the correlation be consistent and replicable. This requires that the approach prove that, under the same method in different circumstances and under different methods in the same circumstances, the correlations will identify the same patterns of neural activity for the very same representees (Yang et al., 2012). This is especially important for various reasons. First, it is important to discard spurious correlations, owing to the evanescence common to patterns of neural activity. Correlation methods can indeed find links between patterns of neural activity and representees that are contingent to the specific conditions of the measure. Thus, it could be that, 2 h later or in some other moment, the correlation would not hold. For example, neural representations can change rapidly due to a number of circumstances, such as training or denervation (Froemke et al., 2007; David et al., 2012; Wymbs and Grafton, 2015). On the other hand, it is important to accept only genuine correlations. If the representation is assumed to be about a representee, then the approach must also show that its candidate for representee correlates with the neural activity under *different tokens of the same representee*. Therefore, to be considered a genuine correlation between a representee and a neural activity, it must hold independent of the situation.

The previous set of criteria subsumes, in my opinion, the commonalities of any account of neural representation shared by both the authors and extant research. It is true that, as discussed previously, there are as many versions of neural representation as there are authors addressing this issue, but my impression is that the proposal I present herein should not dissatisfy many authors. Moreover, I believe that it will be helpful in providing a benchmark against which to assess specific experimental studies that claim to identify and characterize neural representations, and it will also be helpful in identifying how to improve those characterizations in those cases that do not comply with the requirements. I will provide an example of how such an assessment would look when applied to the set of conditions in the two research domains of the survey—that is: place-cell research and MVPA studies. They also provide good example for withstanding scrutiny relative to the definition that I have presented above.

## Probing Mvpa’s Assumptions

Recent years have seen the development of a new set of techniques of analyzing neuroimaging data. Specifically, multivariate pattern analysis (MVPA) tools have been used to map stimuli features into BOLD signals across the brain and vice versa. The ambitious scope of the approach has led authors to use the label of “neural mind reading” in describing such studies. A neural reading MVPA analysis usually involves a three-space model: the stimuli input information, the feature space, and the BOLD signal. A visual stimulus, for instance, is characterized along axes that correspond to the luminance of each pixel, and a natural scene is represented by a single point in the input space. In such a feature space, each axis corresponds to a single feature, and each stimulus is represented by one point in that feature space. Finally, the activity space represents the activation of all the voxels within an ROI (region of interest): the axes correspond to the individual voxels, and ROI’s activation pattern is identified by a unique point in the activity space. In general, the transformation from the input space into the feature space is a non-linear mapping, whereas the transformation from the feature space to the particular BOLD activity, or vice versa, is a linear mapping. In this later case, the mapping involves training a linear classifier that allows mapping multi-voxel activation patterns onto specific stimulus labels.

The MVPA approach has already yielded impressive results, but does MVPA withstand the scrutiny of our operational definition?

### Assessment of MVPA-Based Patterns of Neural Activity

As stated above, the conditions for patterns of neural activity to be considered neural representation are soundness, well-formedness, and self-containment. For **well-formedness**, MVPA research seems to satisfy the condition. It is true that, as I said above, the grammar defining the particularities of well-formedness should be derived from the theory of neural representation supported by the specific approach (in this case, MVPA). However, as stated previously, we still lack such a theory. MVPA has assumed the idea that even if we do not know how representations work, we know they exist. Hence, the task is to reveal them, and MVPA does this in a systematic and formal way that can be considered to be a grammar of sorts by prescribing what the basic constituents of a pattern of neural activity should be and how such constituents are combined to build a particular pattern. Specifically, MVPA departs from the BOLD activity of single voxels by creating multivoxel patterns of activity, and these patterns are then treated as points in a multidimensional space with as many dimensions as voxels there are in the analysis. If we have only two voxels, then a particular pattern can be conceived as a line on a plane based on each voxel’s activity. For more than two voxels, the plane reflects higher-dimensional space. Then, each multivoxel BOLD activity can be transformed in a multidimensional pattern, and all such patterns can be classified in, for example, two different conditions. It is true there are different versions of the grammar employed that, among other things, depends on the type of classifiers—that is, the algorithms classifying the data into conditions. In any event, each version provides its own grammar, with specific rules and constraints, therefore making it possible to assess the well-formedness of its neural representations. Therefore, MVPA can be said to comply with the well-formedness condition in what concerns the *form* of the pattern of neural activity, awaiting an understanding of how such a form is the outcome of the representational encoding.

Next, we address the condition of **self-containment**, that is, the condition that assumes that the pattern should comply with its role regardless of the representee being there. Despite the dearth of studies, there are nevertheless a few that have directly probed this condition. Specifically, the authors of these studies have applied MVPA methods to decode imagined movements (Filimon et al., 2015) and have therefore demonstrated that MVPA patterns are activated in the representee’s absence that overlapping patterns that are activated when the movement is executed. Therefore, even if asserting that MVPA complies with the self-containment condition (for example, proving the contribution to inferential processes) remains relegated to the future, preliminary evidence does suggest that compliance is plausible.

Finally, regarding the **neurally sound** condition, MVPA faces more challenges than it did to meet the previous two conditions. First, the underlying neural characterization of the BOLD signal is still a matter for discussion. In principle, the magnitude of the BOLD signal is associated with the magnitude of underlying neural activation. This has led to the widespread consideration that there is a linear association between BOLD response and the implication of specific neural structures in cognitive processing. However, the BOLD signal actually measures blood oxygenation, which is an indirect measure of neural activity, and the relationship between neural function and the BOLD signal has yet to be characterized in detail. Many aspects of neural activity (e.g., synaptic activity, spiking activity, glial metabolic activity) contribute to the BOLD signal, and the functional implication of the specific neural activity underlying a BOLD signal is not comprehensively known. Therefore, much more precise information about the relationship between the BOLD signal, neural activity and cognitive processing is needed before we can rely on BOLD-data analysis to understand the neural activity being tapped when using MVPA techniques. Likewise, it is still unclear how changes in BOLD signals across time are to be correlated with neural activity. In this sense, the temporal and spatial resolution of the BOLD signal is not adapted to neuronal activity. In the window of time that a BOLD signal is characterized, many relevant neural activities implicating many different neural representations might have taken place without permitting detection by MVPA classifiers. Furthermore, we still do not know at what level (i.e., neuronal, neuronal group, systems) the information relevant to the task is processed, and it could be the case that the measured signal is irrelevant for probing the informational level (Rolls and Treves, 2011). Moreover, it could be that in a single voxel, we could have different levels of informational process and exchange, such that MVPA may only be sensitive to one of them. As Haynes frames it, “Neither the information contained in single voxels nor in ensembles of voxels can be directly related to the information encoded in single neurons. The sampling of neural activity by fMRI voxels is highly indirect and involves the magnetization level of blood as a marker of neural activity, a pooling of many thousand neurons per voxel, and a sluggish and non-linear hemodynamic response” (Haynes, 2015, p. 262). Thus, I believe that any interpretation of MVPA results has to take into account this uncertainty and avoid strong assumptions about its neural import. As Serences and Saproo have written, “MVPA approaches are an extremely powerful tool for determining if there is a difference between activation patterns evoked by experimental conditions. However, the reliance on a weighted pooling of information across many voxels obscures information about exactly how the pattern of underlying neural activity changes as a function of task demands” (Serences and Saproo, 2012, p. 440). The fact remains that MVPA consists of a set of powerful mathematical tools that identify subtle patterns of correlation between huge matrices of numbers. Such an approach is so versatile and productive that one runs the risk of losing sight of what the numbers really measure. Therefore, for the moment, we must say that the jury is still out regarding the neural soundness of the MVPA approach.

### Assessing MVPA-Based Representees

Let us address now the conditions for sanctioning the way MVPA characterizes representee candidates. For the **measurable** and **self-subsistence** conditions, MVPA can be said to comply with both conditions for representees. MVPA methods require that representees be measurable variables that are characterized independently as stimuli features, properties or categories. A simple representee can be, for example, a category, such as “animate objects,” which are to be distinguished from another category, such as “inanimate objects.” It is true that the categorization is made by the scientist, but there are also examples where the stimulus is transformed into a set of physical dimensions. For example, any visual stimulus whatsoever—say, a landscape—may be selected and then transformed into a feature space where the scene is characterized by different dimensions that correspond to the luminance of each pixel, such that the scene is ultimately represented by a single point in the input space. In such a feature space, each axis corresponds to a single feature, and each stimulus is represented by one point in that feature space. In sum, MVPA fully complies with the measurable and self-subsistence conditions.

Regarding the **meaningful** condition, MVPA faces some challenges. For one thing, MVPA is based on a priori hypotheses about the stimuli features that might be represented. Indeed, in the great majority of studies, an MVPA results from a pre-selection by the scientist of the candidate representees, such as when the scientist studies the distinction between two categories (Borst and Theunissen, 1999). Classifiers are tools that classify *what the scientist tells them to classify*—that is, the scientist has already made the decision on what must be classified (which features to select), and how such parameters vary in the chosen stimulus ensemble can lead to different results. Therefore, the representee is not a *given*, but a *chosen*. Even in the case where the stimuli are transformed into a set of dimensions, such as pixel luminance, the scenes to be classified are chosen by the scientist. This has to be taken into account, because classifiers are so powerful that they are capable of finding correlations between nearly any pattern and any feature. Hence, classifiers run the risk of mapping a set of features that have no representational relevance. Scientists can easily choose an arbitrary set (for example, “things that weigh less than 125 g”) and find reliable patterns of activation that correlate with any such features. The risk of inferring spurious representees has been discussed by some authors (Logothetis, 2008; Yang et al., 2012), and the suggestion to fulfill the condition of meaningfulness is to provide as much independent evidence supporting the choice as possible. Therefore, it will be very important for complying with this condition to assure beforehand what is meaningful vs. what is not meaningful for a certain organism and then to confirm via assessment that the MVPA methods do discriminate between the two, with significant correlations for the former but not for the latter. At this moment, this is not a general methodological provision in MVPA studies, although it would be quite easy to incorporate by showing that within a set of meaningful and non-meaningful representees, the applied MVPA solely identified meaningful correlations.

### Assessing MVPA-Based Correlations

Multivariate pattern analysis has many available types of algorithms to establish the correlation between patterns of neural activity and environmental features. The most common are those known as the linear support vector machine (SVM), but there are also others based on nearest neighbor, Bayesian analysis, linear discriminant analysis, and multinomial logistic regression, among others. As I indicated above, the authors seemed to be very flexible in accepting any technique shown to enjoy suitable and robust technical performance. However, as some authors have indicated, it is one thing for a method to technically perform; it is another to assert the scientific relevance of its findings. As one interviewed scientist phrased it, “It is not enough to show that a particular algorithm finds a correlation between a candidate representation and the represented object; we need independent evidence to sanction such a correlation as genuine.” In the case of MVPA, the challenge is to show that the actual algorithm employed is irrelevant with regard to the findings—i.e., that the MVPA approach is, in general, *reliable* enough, regardless of particular algorithms, to find genuine correlations between a particular pattern of neural activity and its representee. This issue of **reliability** has two sides.

On one hand, “reliable” means that different MVPA algorithms should yield the same results with the same datasets; otherwise, the pattern of neural activity corresponding to a particular neural representation would be algorithm-dependent. In such a case, we would have as many patterns as there were algorithms available, which would undermine the reliability of MVPA as a tool to identify neural representations. On the other hand, “reliable” means that MVPA classifiers should identify the same patterns of neural activity in all the instances of its representee; otherwise, MVPA would be context-dependent, and that would imply that the findings could not be reliably interpreted as a marker of particular representee but instead as a marker of the representee-in-context. Regarding the former aspect of reliability, technique-dependence cannot be characterized as black and white. The choice of a particular algorithm depends on a number of factors, beginning with the question we want to answer (e.g., classification or encoding), particularities of the task (e.g., the number of features involved), and the protocol (e.g., high or low number of repetitions), as well as upon many other factors. Therefore, it might not be useful to compare two classifiers in the same circumstances. However, regardless of this caveat, there are already some studies that have begun assessing how different classifiers identify similar patterns of neural activity for particular representees (Mur et al., 2009; Pereira and Botvinick, 2011), with promising results.

Regarding the question of context-dependence, some authors have made observations leading them to argue that MVPA findings are indeed context-dependent. However, this is an empirical question that can only be resolved with further studies. Moreover, it is conceivable that careful experimental designs can provide evidence of context-independent MVPA methods, as some have already shown (Chang et al., 2009).

## Probing Place-Cell Empirical Accounts

After decades of research and numerous studies, place-cell research has demonstrated the existence of some type of association between information in the environment and the rate and/or temporal spike activity of neurons. When an animal moves through the environment, certain neurons preferentially respond upon passing through a specific location. In the specific case of place cells, networks of neurons in the hippocampus show preferential activity in particular spatial locations, or place fields. Such information is so precise that scientists can accurately estimate the rat’s position in space by observing the rate coded output of just 50 simultaneously recorded place cells. Numerous studies have confirmed and extended these observations (for reviews, see Moser et al., 2015; Schiller et al., 2015). Other developments of place-cell research include the discovery of what is now known as “grid cells” (medial entorhinal neurons that fire when rats occupy a spatially periodic array of locations), hippocampal neurons that process head direction and border cells, as well as other types of cells with spatial firing characteristics.

The success stories of these discoveries suggest that the hippocampus supports navigation via some sort of internal representation of space, as O’Keefe and Nadel (1978) originally suggested. Can this representational assumption for place-cell research withstand the previous criteria for neural representation?

### Assessment of Place-Cell Based Patterns of Neural Activity

Regarding the **well-formedness** requirement, place-cell research faces, as in the MVPA case, the challenge of lacking a theory of neural representation. However, as we have also seen in the case of MVPA, place-cell research has overcome this handicap by assuming that representations exist and that they can be identified through its methodology. Moreover, the methodology is so well established and formalized that it can be considered a grammar, as I have described above: it prescribes what the basic constituents of a pattern of neural activity should be and how such constituents are combined to build a particular pattern. In this sense, place-cell research apparently provides a strong and well-established characterization of the features of spike activity that the brain seems to use as an encoding scheme. On one hand, we have the neuronal firing rate, that is, the average number of spikes per unit time that provides a rate code; on the other hand, we have the temporal code, which is based on the precise timing of single spikes. However, the fact remains that the jury is still out on the issue of whether the brain uses rate coding, temporal coding or a combination of both (Masuda and Aihara, 2007). Moreover, discussions even address the possibility that there may not be a difference between the temporal and rate codes (Shinomoto and Koyama, 2007). Furthermore, it is not even clear how rate/temporal code is associated with a certain memory/representation. For one thing, reactivating the neurons of a hippocampal ensemble associated with a context can be sufficient to trigger the retrieval of a memory, indicating that it is not necessary to activate the rate/temporal firing patterns originally associated with the context (Smith and Bulkin, 2014). Therefore, we can say that place-cell research offers the fundamentals of a neural-code grammar, even if it is still a work in progress.

Regarding the condition of **self-containment**, I believe that place-cell research faces a real challenge. Most place-cell representational assumptions are based on patterns of neural that are active *only* when the supposed representee is present—that is, when animals are *in* the place that is supposed to be represented. In fact, place-cell representations seem to represent only *current* location. There are indications, though, that sequences of spatial firing during exploration were shown to be replayed during rest or sleep subsequent to the behavioral experience, as if those patterns were stored in the hippocampal network during exploration and retrieved later in offline mode (Jadhav et al., 2012; Jahnke et al., 2015; Ambrose et al., 2016; Deng et al., 2016; Rothschild et al., 2016). It is thus beginning to be reasonable to think that neural representations for places can be activated when necessary. In sum, although the condition of self-containment has not been fully met by place-cell research, there are indications that it could eventually be complied with.

Finally, place-cell can be said to represent a **neurally sound** approach. The recording of electro-physiological activity is one of the oldest and most widely used techniques in neuroscience. Using microelectrodes close to the cell surface, researchers have been able to record the voltage changes outside the cell, acquiring information about the number of neuronal action potentials, or spikes, generated in a unit of time. This provides direct evidence of neuronal activity; it is therefore difficult to imagine an approach with a stronger foundation. However, we must not forget that, as with MVPA, place-cell research lacks a general theory of neural representation; thus, we still do not know in virtue of what a particular pattern of neural activity is the representation of a particular environmental feature. So, at most, what we can say is that the approach is neurally justified, although it is still awaiting to ground its basics in a theory of neural representation.

### Assessing Place-Cells’ Representees

As for representees, place-cell research seems to comply fully with all three criteria. Spatial location is a **measurable** and **self-subsistent** magnitude, and it is also a fundamental and **meaningful** aspect of living beings.

However, this compliance may only be a misperception. It has been well-known from the very beginning of hippocampal research that place cells are sensitive to non-spatial cues. The fact is that place cells have been found to be sensitive to many non-spatial features (Wood et al., 1999), including odors (Komorowski et al., 2009), tactile inputs (Itskov et al., 2011), tones (Itskov et al., 2012), timing, (Manns et al., 2007; MacDonald et al., 2011; Kraus et al., 2013; Wikenheiser and Redish, 2015; Ranganath and Hsieh, 2016), rewards (Markus et al., 1995; Wikenheiser and Redish, 2011), expectations (Skaggs and Mcnaughton, 1998), goals (Breese et al., 1989), and other motivational states of the individual (Kennedy and Shapiro, 2009), as well as to higher-order features such as trajectories (Shapiro et al., 2006; Griffin and Hallock, 2013), task contingencies, such as the strategy to solve a task (Eschenko and Mizumori, 2007), and even the type of active/passive role played by the individual (Terrazas et al., 2005).

Some authors have addressed how non-spatial sensitivity could affect the spatial representational hypothesis of the hippocampus, by appealing to second-order functions, such as context, sequences or predictions. Since the beginning of hippocampus research, it was quite clear that hippocampal cells were sensitive to context (Eichenbaum et al., 2012), understood very generally as a set of background elements in the environment that determine the meaning of a particular situation. For example, place cells change their activity patterns in response to changes in context, regardless of the activity’s occurring in the same location (for a review, see Smith and Bulkin, 2014). According to a contextual processing interpretation, spatial cues, although critical, would only be one component among several that define context. Another explanation, not necessarily incompatible, is that the hippocampus might use spatial and non-spatial information to encode sequences of events. Computational models have suggested (Rolls and Kesner, 2006), for example, that place cells could be a time-point marker in a sequence. Experimental evidence supports this hypothesis, showing that place-cell activity during navigation activity is spontaneously emitted independent of immediate spatial cues as steps in a sequence (Johnson and Redish, 2007). Based on a similar sort of evidence, there are authors who suggest that place cell activity would play a central role in prediction (Buckner, 2010; Lisman and Redish, 2011). For instance, the fact that place cells do not simply fire based on inputs that specify a single location but rather are triggered by inputs to earlier locations that anticipate them can be understood as an example of sequential processing as well as a way of predicting future events based on past events (Diba and Buzsáki, 2007; Pastalkova et al., 2008; Pfeiffer and Foster, 2013, 2015a,b). This idea fosters the intuitive notion that the role of memory has an adaptive function and is related to the present and future of the animal (Suddendorf and Corballis, 2007), rather having the function of preserving the past. Schacter and Addis (2007) write, “Simulation of future episodes may require a system that can draw on the past in a manner that flexibly extracts and recombines elements of previous experiences—a constructive rather than a reproductive system” (Schacter and Addis, 2007, p. 774).

The bottom line is that any of these new interpretations of hippocampal function involve a challenge to the notion of representee. Neither context, nor sequences nor prediction can be seen to be a *self-subsistent* element of the environment. Rather, they are constructs that do not exist independent of the specific interaction of the animal with the environment in the precise moment of the interaction. Moreover, they do not seem to be elements to be represented—they are not informational states that map, encode or refer—but instead are constructs that address the situation. All of this challenges the notion of neural representation in itself, upon which I will elaborate below. For the moment, we should conclude that converging evidence from place-cell research suggests non-compliance with the self-subsistence condition for representees.

### Assessing Place-Cell Representational Relations

Place-cell research is recognized as one of the best examples of well-established correlation between an environmental component, a spatial location, and a pattern of neural activity. Indeed, place-cell researchers have systematically shown the strong relationship between a certain pattern of spike activity in some neurons and a specific location within the active environment. In this sense, there exists no other scientific domain wherein the representational relation between an activity in the brain could be said to be more consistent and replicable, that is, **reliable**.

## Discussion

Based on the results of the survey and the neuroscientific literature, I have outlined here a set of conditions that researchers seem to require for accounts of neural representation. For patterns of neural activity, the conditions are neural soundness, well-formedness, and self-containment. By “neurally sound,” I posit that the proposed approach should have a justifiable theoretical basis upon which to support the claim that the variables measured by the approach (a BOLD signal and spike train in the selected examples) correspond to the neural correlates of a candidate representee. “Well-formedness,” in turn, refers to the rules and constraints that should underlie the characterization of patterns of neural activity. Finally, the “self-containment” condition refers to the requirement that patterns of neural activity should show the capacity to substitute the representee in the internal workings of the brain, without the representee being present. For representees, the proposal presented here suggests that representational accounts should select only meaningful, self-subsistent and measurable representees from the set of all possible segmentations of the active environment. Finally, the methods for establishing the relation between the representation and the representee should be reliable—that is, correlations should be context- and technique-independent.

I have used this set of conditions to assess extant MVPA and place-cell research (see **Table 1** for a summary of the assessment). I have shown that MVPA satisfies, at least partially, the conditions of well-formedness and self-containment for the patterns of neural representation, while still failing to fully meet the challenges of neural soundness. MVPA still needs to prove, in turn, that it is discriminative enough in relation to identifying only meaningful representees, and it also must show that it is context- and technique-independent. We have also seen that place-cell research does partially comply with the neural soundness condition for patterns of neural activity. It must also seek to meet the well-formedness condition by establishing a grammar for the neural code, and it also remains to be seen whether it will satisfy the self-containment condition. Concerning the criteria for representees, place-cell research seems to fully satisfy the measurable, meaningful and self-subsistent conditions. Finally, place-cell methods of establishing the relation between patterns of neural activity and representees fully complies with the reliability criterion. We can conclude that extant research on representations partially satisfy the conditions for neural representations and that they would only need more evidence, better neurophysiological understanding, and specific neurobiological and conceptual constraints.

**Table 1:** Criteria for neural representation and its compliance by research domains.

	Place-cell research	MVPA
**Neural activity criteria**		
Well-formedness	In process	Partially satisfied
Self-containment	In process	Partially satisfied
Neural soundness	Partially satisfied	In process
**Representee’s criteria**		
Measurable	Satisfied	Satisfied
Self-subsistence	Satisfied	Satisfied
Meaningfulness	Satisfied	In process
**Representational-relation’s criteria**		
Reliability	Satisfied	Satisfied

A possible objection to the previous analysis may be the degree to which it can be generalized, as I only apply the analysis to two research domains: place-cell and MVPA studies. While I cannot prove my analysis can be generalized to all the domains where neural representation is used, I believe that there are indications that this could be the case. For one thing, I intentionally chose two of the more methodologically and theoretically distant domains in neuroscience, place-cell and MVPA, so as to examine the two more distant uses of the notion of “neural representation.” Place-cell settings function at the level of cell activity, while MVPA focus on patterns of brain activity that encompass the whole brain. Moreover, place-cell research associates the animal’s real behavior with direct electrophysiological data; in contrast, MVPA applies multivariate statistical tools to connect stimuli features with indirect measures of brain activity. However, what place-cell and MVPA research have in common is, namely, processing environmental activity in order to carry out cognitive processes that are connected to behavior, which is something that is shared by many other domains in neuroscience, from animal behavior, up to the more sophisticated cognitive processes of humans. The fact is that the brain does what it does, regardless of the different ways we have of studying it; hence, the explanation of its function cannot be technique-dependent. The notion of neural representation, even if analyzed in specific domains, must possess general and shared properties that concern all levels of analysis. Therefore, I believe the analysis presented here can be generalized in the sense that it can be applied to any domain where the notion of “neural representation” is used.

Moreover, the analysis presented here has opened an intriguing possibility that may have interesting consequences for the notion itself. A careful analysis of place-cell multidimensional sensitivity presents a serious challenge not only to the evaluation of place-cell representees but also to the notion of neural representation in general. Let me explain. During decades of research, the simple representational model of place-cell studies has been based on the assumption that the hippocampus was involved in processing a spatial map of the animal’s environment. This hypothesis has been favored because location is the most informative and temporally stable feature in the environment; therefore, it is highly predictable (Ranganath, 2010; Eichenbaum, 2015; Zucker and Ranganath, 2015). As some authors note (Eichenbaum, 2015), place-place cell research belongs to the neuroscientific tradition wherein the aim has been to identify a single “trigger feature” of each neuron, and then model a circuitry that constructs networks of information processing from simple to more complex coding features. Such a model is based on a hierarchy wherein the brain reconstructs representations in successive stages of processing. A close analysis of the contextual factors involved in place cell processing suggest that the spatial representational perspective of the hippocampus is too simplistic (Eichenbaum et al., 1999). Research has shown that in realistic contexts, neurons throughout the hippocampus have very complex firing properties that challenge the explanatory power of these simple trigger features for the representation of real-life experiences. The fact is that when only single-stimulus dimensions are presented, neurons are impressively selective to a specific representee, but in natural contexts, place cells have highly complex firing patterns that reflect a mixed selectivity to multiple dimensions of ongoing perception, cognition, and behavior. As we have seen above, converging evidence shows that place cells are sensitive to a diverse range of cues, including first-order spatial and non-spatial information, as well as higher-order processing, such as for tasks, goals, or rewards (Eichenbaum et al., 1999). Moreover, under appropriate conditions, prospective activity correlates are detached from stimuli in the immediate environment and the current spatial behaviors of the animal (Buckner, 2010). According to Eichenbaum (2015, p. 680), “this course of observations challenges as too simplistic the approach in which single spatial trigger features are combined in models to perform navigational calculations of the ‘inner GPS’.” According to some authors, acknowledging this situation demands a paradigm shift, or as Eichenbaum have phrased it, “suggests a theoretical revolution underway” (Eichenbaum, 2015, p. 680).

I will now examine how this shift affects the notion of representation. Let us consider the contextual, sequential and predictive functional interpretations of place-cell activity that we have examined above. Context is generally understood, in the area of hippocampal research, as any situation defined by a coherent set of features occurring within a particular environment (Smith and Bulkin, 2014). On the basis of this interpretation, place cells seem to encode contexts that are defined by a variety of environmental (e.g., location), cognitive (e.g., task dimensions) and motivational (e.g., goals) factors directly related to the situation in which the individual is involved. Such elements would take the form of a coherent network that would change as a whole in relation with a particular situation, rather than in a piecemeal fashion for each feature (Smith and Mizumori, 2006). In turn, researchers in hippocampal research understand “sequence” to mean any succession of events that is correlated by place-cell activity at different time points. Several studies have now shown that a sequential organization is actively present in hippocampal networks (Shapiro et al., 2006; Pastalkova et al., 2008; Pfeiffer and Foster, 2013). Finally, some authors have proposed that the processing of sequences could also be seen as a way of predicting the course of events according to present context and past experiences (Lisman and Redish, 2009, 2011; Wikenheiser and Redish, 2014).

My view is that any of the previous hypotheses constitutes a direct challenge to the classical notion of representation by making the classical view useless for understanding what place cells actually do. As I mentioned above, a representation involves a coding, mapping or referring relation between some neural activity and some environmental component. Nevertheless, contexts, sequences or predictions do not seem to be descriptions, maps, or copies of the environment as such. Moreover, they are not self-subsistent elements of the environment, because they do not exist independent of the ongoing animal-environment interaction. Location, for example, does not seem to be treated by the hippocampus as a re-presentation of the environment but as a spatial marker related to the contingencies of the situation; it is one of many dimensions in multidimensional processing that involves spatial features, but it also taps into the motivational state of the individual, the features of the task at hand, the history of similar interactions and many other informational elements relevant for the individual in such a situation. Contexts, sequences or predictions are better understood as complex information states that integrate different sources of information, external and internal to the animal, whose function seems to be structuring the interaction of the animal with the environment in that particular situation. Therefore, the model of representation of a neural activity engaged in mapping, copying or referring to some environmental element cannot characterize the contextual, sequential or predictive processing; the hippocampus seems to be doing something not specifically representational with the information.

Eichenbaum et al. (1999), Lisman and Redish (2009), Vilarroya (2012, 2014), Zucker and Ranganath (2015) have already suggested a possible theoretical framework that can be used to account for the hippocampal function. The shift suggested by these authors can be summarized as changing the focus on the hippocampus function from *representing* the environment to *guiding action through anticipation*. Instead of mapping or encoding processes, the hippocampus might be set to control the contingencies of a situation by efficiently integrating environmental, cognitive and motivational information into a processing state ready to guide the individual in that particular situation. In other words, the hippocampus could be better seen as a system with the function of providing a dynamic state space where “the set of probable events and contingencies” (Zucker and Ranganath, 2015, p. 700) linked to a particular situation could be managed. As Lisman and Redish indicate, “The hippocampus is not involved in the generation of simple expectancies, as used in typical instrumental learning tasks (…), but it is involved in accommodating complex changes in contingencies (as in contingency degradation tasks; …). This ability to deal with complexity might allow the hippocampus to combine information to produce a prediction of events that never happened. From a cognitive perspective, it seems clear that both simple recall of memories and constructive processes take place” (Lisman and Redish, 2009, p. 1199). Moreover, they consider that such activity is used by the animal to guide behavior. In short, if we understand a “situation” as a particular temporal, spatial and meaningful context in which an animal is involved that is set in dynamic framework where past, present and future events are integrated, then the “content” of place-cell neural activity would correspond to the integration of environmental, motivational and cognitive dimensions into an informational density that anticipates events and guides the animal in that very situation.

Accordingly, instead of computing “representational” processes, the hippocampus would be better described as computing “situational” processes. The term “situational” has already been used in the sense of a cognitive construction meaningful to the animal only in the situation in which it is involved and probably related to helping the animal to address it (Lisman and Redish, 2009; Schiller et al., 2015). Hence, “situational” may be an appropriate means of labeling this approach, although its use does not intend to exclude all situational processing from the classical notion of representation.

It is important to note, however, that the hypothesis suggesting that the hippocampus processes situations instead of elements of the environment is not exclusive. It could very well be that the hippocampus has overlapping modes of processing and that one mode could represent objective coordinates of the spatial layout of the animal. This is obviously an empirical question. Moreover, the situational approach might not have any relevance outside the hippocampus; thus, it might not affect MVPA research. The point is that place-cell research evidence so far might be better understood within the situational approach. In any event, the suggestion of a situational model obviously requires further investigation and development.

## Conclusion

As presented above, neural representation is a central and non-trivial notion in extant neuroscience. Authors extensively use the notion, putting important explanatory weight on it. However, no agreed benchmark against which to assess specific theoretical and empirical claims exists. Based on the survey and previous literature, I have outlined a set of properties with which an operational account of neural representation should comply. The idea is that one could use such properties as a way of assessing a particular claim of representation. However, the evaluation of place-cell research under such criteria raises the intriguing possibility that the neural activity of place cells might be better understood at present within a non-representational framework. The representational scheme based on the idea that neural activity encodes, maps or refers to self-subsistent, meaningful and measurable features of the environment might not be the best way to construe the role of place cells in hippocampal function. Instead, a situational approach, based on the integration of spatial and non-spatial information with the aim of creating a state space where the contingencies of a situation are processed, seems to be more useful for understanding place-cell activity. The challenge now is to explore and develop this situational approach.

## Author Contributions

OV conceived the article, carried out the survey and its analysis, and wrote all the versions of the manuscript.

## Conflict of Interest Statement

The author declares that the research was conducted in the absence of any commercial or financial relationships that could be construed as a potential conflict of interest.
